# Expression and extracellular release of a functional anti-trypanosome Nanobody^® ^in *Sodalis glossinidius*, a bacterial symbiont of the tsetse fly

**DOI:** 10.1186/1475-2859-11-23

**Published:** 2012-02-15

**Authors:** Linda De Vooght, Guy Caljon, Benoît Stijlemans, Patrick De Baetselier, Marc Coosemans, Jan Van Den Abbeele

**Affiliations:** 1 Department of Biomedical Sciences, Unit of Medical Entomology, Institute of Tropical Medicine Antwerp, Antwerp, Belgium; 2 Department of Biomedical Sciences, Unit of Veterinary Protozoology, Institute of Tropical Medicine Antwerp, Antwerp, Belgium; 3 Unit of Cellular and Molecular Immunology, Vrije Universiteit Brussel, Brussels, Belgium; 4 Laboratory of Myeloid Cell Immunology, VIB, Brussels, Belgium

**Keywords:** *Sodalis glossinidius*, Symbiont, *Glossina*, Paratransgenesis, Expression, Nanobody, Functional

## Abstract

**Background:**

*Sodalis glossinidius*, a gram-negative bacterial endosymbiont of the tsetse fly, has been proposed as a potential *in vivo *drug delivery vehicle to control trypanosome parasite development in the fly, an approach known as paratransgenesis. Despite this interest of *S. glossinidius *as a paratransgenic platform organism in tsetse flies, few potential effector molecules have been identified so far and to date none of these molecules have been successfully expressed in this bacterium.

**Results:**

In this study, *S. glossinidius *was transformed to express a single domain antibody, (Nanobody^®^) Nb_An33, that efficiently targets conserved cryptic epitopes of the variant surface glycoprotein (VSG) of the parasite *Trypanosoma brucei*. Next, we analyzed the capability of two predicted secretion signals to direct the extracellular delivery of significant levels of active Nb_An33. We show that the pelB leader peptide was successful in directing the export of fully functional Nb_An33 to the periplasm of *S. glossinidius *resulting in significant levels of extracellular release. Finally, *S. glossinidius *expressing pelBNb_An33 exhibited no significant reduction in terms of fitness, determined by *in vitro *growth kinetics, compared to the wild-type strain.

**Conclusions:**

These data are the first demonstration of the expression and extracellular release of functional trypanosome-interfering Nanobodies^® ^in *S. glossinidius*. Furthermore, *Sodalis *strains that efficiently released the effector protein were not affected in their growth, suggesting that they may be competitive with endogenous microbiota in the midgut environment of the tsetse fly. Collectively, these data reinforce the notion for the potential of *S. glossinidius *to be developed into a paratransgenic platform organism.

## Background

Tsetse flies (*Glossina *sp.) are medically important, viviparous dipterans that transmit *Trypanosoma *spp. parasites responsible for human sleeping sickness and Animal African trypanosomiasis. This biological transmission is based on a complex developmental cycle that these protozoan parasites have to go through in the tsetse fly alimentary tract and mouthparts (*Trypanosoma congolense*) or salivary glands (*T. brucei *sp.). Similar to other blood feeding insects, tsetse flies rely on bacterial symbionts to acquire nutrients that are insufficiently present in their diet and which they are unable to synthesize themselves. *Sodalis glossinidius *is a maternally inherited gram-negative bacterial endosymbiont of the tsetse fly that can be found both inter- and intracellularly in the tsetse fly midgut, muscle, fat body, milk glands, and salivary glands [1]. Given the close proximity of *S. glossinidius *to the different insect tissues where trypanosome parasites reside and the fact that it is one of the few insect symbiotic bacteria that can be cultured and genetically modified *in vitro *[1-3], *S. glossinidius *is considered as a potential *in vivo *drug delivery vehicle to control *T. congolense *and *T. brucei *development in the fly. This strategy involving the use of bacterial symbionts to express foreign proteins, designed to block pathogen transmission, is often referred to as paratransgenesis [4] and has been developed and proposed to combat different insect-borne animal and human diseases [5-7]. The control of the trypanosome parasite in the tsetse fly using paratransgenic technology requires the identification and characterization of gene products that interfere with trypanosome development without reducing the fitness of the tsetse host or its symbiont.

To date, genes involved in insect immunity have attracted most attention as potential antitrypanocidal effectors. In *Glossina morsitans morsitans*, the host defense genes *attacin *and *defensin *are up-regulated in response to trypanosome infection [8-10] and antitrypanosomal activity has been shown for the recombinant attacin protein [11]. Another potential effector protein is an antimicrobial host defense protein produced by bovine neutrophils with trypanocidal activity, BMAP-27 [12]. Indeed, this bovine myeloid antimicrobial peptide has been shown to exhibit high toxicity towards two major life cycle stages of African trypanosomes (bloodstream-form and procyclic-form) However, to date none of these peptides have been successfully expressed in the tsetse fly bacterial symbiont *S. glossinidius*.

The identification of monoclonal antibodies (mAbs) that recognize parasite surface proteins, and their subsequent expression as single chain antibody gene fragments (ScFv), provides an alternative as potential antipathogenic effectors. The feasibility of expressing ScFvs in bacterial symbionts that retain their functional activities has been demonstrated in *Rhodococcus *[13] and *Pantoea agglomerans *[7]. However, due to their bulky size (30 kDa range) and complex architecture mAbs are often prone to aggregation and reduced affinity [14,15].

Nanobodies^® ^(Nbs) represent the smallest known intact antigen-binding fragments derived from heavy-chain only antibodies (HCAbs), devoid of light chains, naturally occurring in *Camelidae *and sharks [16-18] (reviewed by [19]). Due to their ability to target unique epitopes that are less well targeted by conventional antibodies, Nbs are currently of high research interest for various pharmaceutical applications, including diagnosis and drug delivery [20,21]. Because of their superior intrinsic properties, e.g. small size (13-15 kDa), strict monomeric behavior and high *in vitro *stability [17,22] Nbs are efficiently produced in micro-organisms such as *Escherichia coli *[23] and therefore show high potential as effector molecules in the paratransgenesis approach. Nbs directed towards distinct regions of the variant-specific surface glycoprotein (VSG), abundantly present on the surface of bloodstream trypanosomes, have already been identified, targeting VSG epitopes that are inaccessible on live trypanosomes for larger antibodies [24]. An essential aspect for a successful symbiont-based paratransgenesis approach is the active release of the effector molecules to the inner insect environment for efficient targeting of the pathogen. However, few studies have focused on the export of heterologous proteins to the periplasmatic and/or outer environment of *S. glossinidius*.

In this study, we aimed to investigate the potential of *S. glossinidius *to express functional Nbs without interfering with cell viability. Secondly, we evaluated the capability of two independent secretion signals, predicted to be involved in different protein secretion pathways, to deliver the effector proteins to the extracellular environment. We show that Nb_An33, recognizing a VSG epitope on *Trypanosoma brucei *[24] was expressed by *S. glossinidius*. Furthermore, we demonstrated that the pectate lyaseB (pelB) signal peptide from *Erwinia carotovora *is able to direct the export of fully functional Nb_An33 to the periplasm of *S. glossinidius *resulting in significant levels of extracellular release. Importantly, *Sodalis *strains that efficiently released the effector protein were not affected in their growth, suggesting that they may be competitive with endogenous microbiota in the midgut environment of the tsetse fly.

## Results

### Expression of Nb_An33

**Figure 1:**
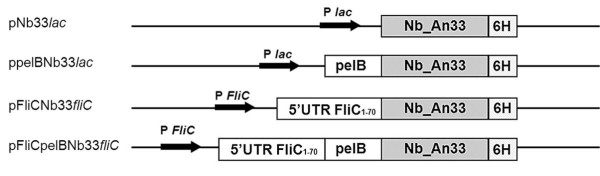
**Schematic presentation of the gene constructs used in this study**. Lines represent untranslated DNA regions, filled bars the coding region of *Nb_An33 *and open bars the coding regions of the respective secretion signals. All gene constructs contain a 6xhis tag (6H) at the carboxy terminal end for purification purposes. P*lac *and P*FliC *indicate the *lac *promoter and the promoter region of the *S. glossinidius fliC *gene respectively. 5'-UTR indicates the 5' untranslated sequence of *fliC *including the *fliC *promotor. FliC_1-70 _refers to the size in amino acids of the fragment of the *S. glossinidius *FliC protein containing the predicted signal sequence.

**Figure 2:**
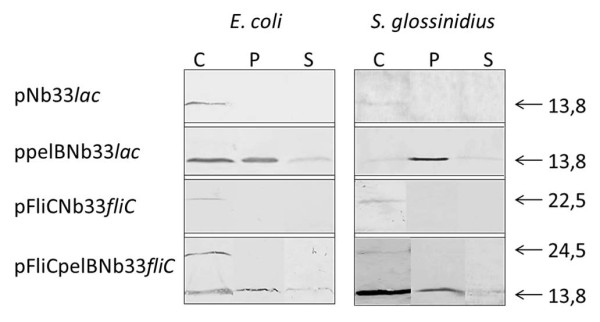
**Qualitative analysis of intracellular (cytoplasmic and periplasmic) and extracellular Nb_An33 fusion proteins expressed from *E. coli *(left panel) and *S. glossinidius *(right panel) harboring the different expression plasmids**. *E. coli *and *S. glossinidius *samples were not normalized for cell density as these two host cells reach different cell densities at early stationary phase. The localization of the expressed Nb-An33 fusions were analyzed by immunoblotting of cytoplasmic (C) and periplasmic (P) fractions and medium supernatant (S) using an anti-His antibody (1:1000 Serotec) for detection. Presented data are representative for at least three independent experiments. Molecular size markers in kDa are indicated on the gels.

We examined the capability of *Sodalis glossinidius *to express/export Nb_An33 through the use of expression plasmids harboring the coding sequence for *Nb_An33 *fused to two independent secretion signals, one of which is native to *S. glossinidius*. A schematic presentation of the structure of the fused genes is shown in Figure 1. For the first secretion construct, *Nb_An33 *was fused to the *pelB *leader sequence. As *S. glossinidius *is devoid of a *lac *repressor, the presence of the *lac *promoter resulted in a constitutive expression of pelBNb_An33. The second secretion signal was that for the flagellin protein FliC of *S. glossinidius*, one of the most abundant proteins on the bacterial surface and the major component of the flagellum [25]. Here, the construct contained the promoter and the 5'untranslated region (UTRs) of the *S. glossinidius FliC *gene which according to Majander *et. al*. [26] facilitates the extracellular secretion of polypeptides via the type III pathway in *E. coli*. The expression of the different Nb_An33 constructs was examined in both *E. coli *and *S. glossinidius*. The pelB leader resulted in the extracellular release of Nb_An33 from *S. glossinidius *and *E. coli *as shown by Western blot analysis (Figure 2). Here, the Nb_An33 is present as a 13.8 kDa protein both in the periplasmic fraction and cell-free culture supernatant of both bacterial species. As this size is identical with the Nb_An33 that was expressed without any secretion signal, it confirms the correct cleavage of the pelB leader peptide during periplasmatic transport. Moreover, the Nb_An33 devoid of the secretion signal accumulated exclusively in the cytoplasm. The FliC-Nb_An33 fusion protein was expressed in the cytoplasm of *S. glossinidius *and *E. coli*, however in neither species was the fusion protein exported to the periplasm nor extracellular medium. Figure 2 shows that the fusion protein remained within the cells as a 22.5 kDa protein, corresponding with the molecular weight of the fusion construct. Finally, an expression plasmid was constructed containing both *pelB *and *FliC *secretion signals fused to *Nb_An33 *and under the control of the *FliC *promotor, i.e. pFliCpelBNb33*fliC*. Here, Nb_An33 could be detected as a 13.8 kDa protein both in the periplasm and extracellular medium of *E. coli *and *S. glossinidius*. Interestingly, immunoblotting of cytoplasmic fractions shows that two forms of the FliCpelB-Nb_An33 fusion protein could be detected: Nb_An33 (13.8 kDa) and Nb_An33 coupled to both pelB and FliC secretion signals (24,5 kDa). The expression and localization of the Nb_An33 for the different expression constructs are summarized in Table 1.

**Table 1:** Summary of the gene constructs expressed in *S. glossinidius*, cellular localization of the resulting polypeptides and characteristics of the recombinant *S. glossinidius *strains i.e., culture doubling time (mean ± SD) and protein yields at day 8 (mean ± SD).

Expressed gene construct	Cellular localization	Culture doubling time	Protein yield (day 8) (ng/ml of culture)	
			I	E
*Nb_An33*	C	8.74 h ± 0.1	BDL	BDL
*pelBNb_An33*	C, P, S	7.87 h ± 0.04	26.54 ± 3.6	111.05 ± 114
*FliCNB_An33*	C	8.92 h ± 0.15	BDL	BDL
*FliCpelBNb_An33*	C, P, S	7.80 h ± 0.14	10.61 ± 0.9	46.97 ± 6.3

Cellular localization; C, cytoplasmic; P, periplasmic and S, supernatant. Protein yield; I, intracellular; E, extracellular. BDL, Below detection limit.

### Growth rates

**Figure 3:**
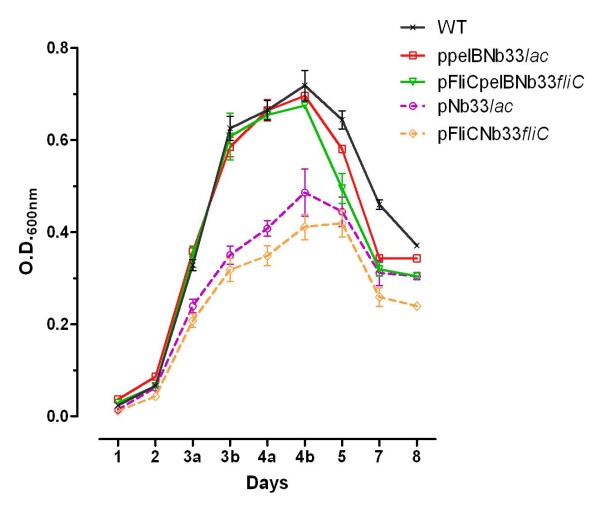
**Growth curve analysis of *S. glossinidius *expressing and/or secreting recombinant Nb_An33**. The error bars show the ± SD of two biological replicates. Samples were taken every 24 h except during exponential growth (day 3 and 4), 2 samples/24 h were taken (a and b).

In order to determine the overall effect of the Nb expression and extracellular release on the bacterial fitness, we examined the growth rates of *S. glossinidius *strains harboring the various expression constructs and compared them to the WT strain (Figure 3). Under standard microaerophilic conditions *S. glossinidius *divides very slowly, however they can be grown under oxidative stress once cultures have reached a cell density sufficient to ensure activation of the oxidative-stress response regulated via a quorum-sensing mechanism (OD_600_: 0.03; [27]). Therefore, cultures were grown without shaking for the first 48 h and then transferred to a shaking incubator. The growth curves of the *S. glossinidius *strains periplasmatically expressing Nb_An33 (*S. glossinidius*:p*pelBNb33lac *and *S. glossinidius*:p*FliCpelBNb33fliC*) were indistinguishable from the WT strain. However, *S. glossinidius *strains expressing Nb_An33 cytoplasmatically (*S. glossinidius*:p*Nb33lac *and *S. glossinidius*:p*FliCNb33fliC*) showed a lower maximum density as compared to the WT strain (Figure 3). These results are consistent with the calculated cell population doubling time of the different strains during the exponential growing phase of the culture. The doubling times (in hours) for the Nb_An33 exporting strains are identical with that of the WT strain whereas the Nb non-exporting *Sodalis *strains demonstrated an elevated doubling time: WT, 7.75 h ± 0.07; pNb33*lac*, 8.74 h ± 0.1; ppelBNb33*lac*, 7.87 h ± 0.04; pFliCNb33*fliC*, 8.92 h ± 0.15; pFliCpelBNb33*fliC*, 7.80 h ± 0.14. These data indicate that intracellular expression of Nb_An33 has an inhibitory effect on growth compared to the WT strain, while secreting strains are not affected in their growth.

### Quantitation of extracellular Nb_An33

**Figure 4:**
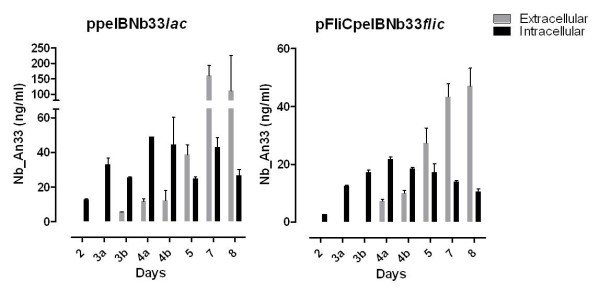
**ELISA based Nb_An33 quantitation using a 6 × His tag specific detection antibody in order to determine the intra- and extracellular nanobody concentration produced by *S. glossinidius *harboring pelBNb33*lac *and FliCpelBNb33*fliC *plasmids at selected time points**. Samples were taken every 24 h except during exponential growth (day 3 and 4), 2 samples/24 h were taken (a and b). Values are presented as ng recombinant protein per ml culture medium. The error bars show the ± SD of two biological replicates. Presented data are representative for two independent experiments.

Nb_An33 expression and release was quantified by measuring the concentration of active Nb_An33 in the whole cell fraction and supernatant using enzyme-linked immunosorbent assay (ELISA). Extracellular and intracellular concentrations of Nb_An33 produced by *S. glossinidius *harboring the pelBNb33*lac *and FliCpelBNb33*fliC *plasmids were analyzed at different time points during bacterial growth over a 8-day period and results are shown in Figure 4. During logarithmic growth (day 3) of the pelBNb33*lac *harboring strain, a release efficiency of 20% was estimated, calculated as the percentage of the Nb present in the culture medium on the total amount of expressed Nb. The release efficiency increased to 25% during stationary phase, i.e. day 4. During this stage of the growth curve (day 1- day 4) no significant cell death occurred as measured by the Live/Dead BacLight bacterial viability test (data not shown). After the cells reached saturation, a drastic increase of active extracellular Nb_An33 was measured from 60% of total protein at day 5 to 80% at day 8. Here, active Nb_An33 accumulated in the culture medium of *S. glossinidius *expressing pelBNb33 at concentrations of 160 ng/ml. The release efficiency of *S. glossinidius *harboring the FliCpelBNb33*fliC *plasmid showed a similar pattern as the pelBNb33*lac *harboring strain with an efficiency of 35% and 81% at day 4 and 8 respectively. However, the amount of active intra- and extracellular Nb_An33 produced by *S. glossinidius *expressing *FliCpelBNb33 *was lower compared to the *pelBNb33 *expressing strain. Given that these concentrations were determined by a VSG-binding ELISA these results indicate that the Nb_An33 expressed by recombinant *S. glossinidius *is functional in terms of antigen-binding.

### Nb_An33 released by *Sodalis glossinidius *targets live trypanosomes

**Figure 5:**
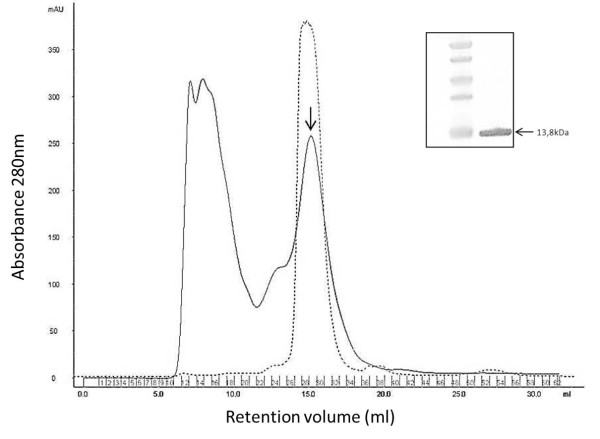
**Size-exclusion chromatography profile (solid line) of the Ni-NTA eluted periplasmic extract from *S. glossinidius *expressing *FlicpelBNb33 *loaded onto a Superdex-75 (10/30) column using PBS as running buffer**. Affinity purified *S. glossinidius *Nb_An33 eluted at the positions indicated by the arrow. The dotted line represents the elution pattern of Nb_An33 purified from *E. coli*. Eluted fractions containing *S. glossinidius *Nb_An33 were pooled and purity was evaluated by SDS-PAGE gel electrophoresis and anti-His Western blot (inset).

**Figure 6:**
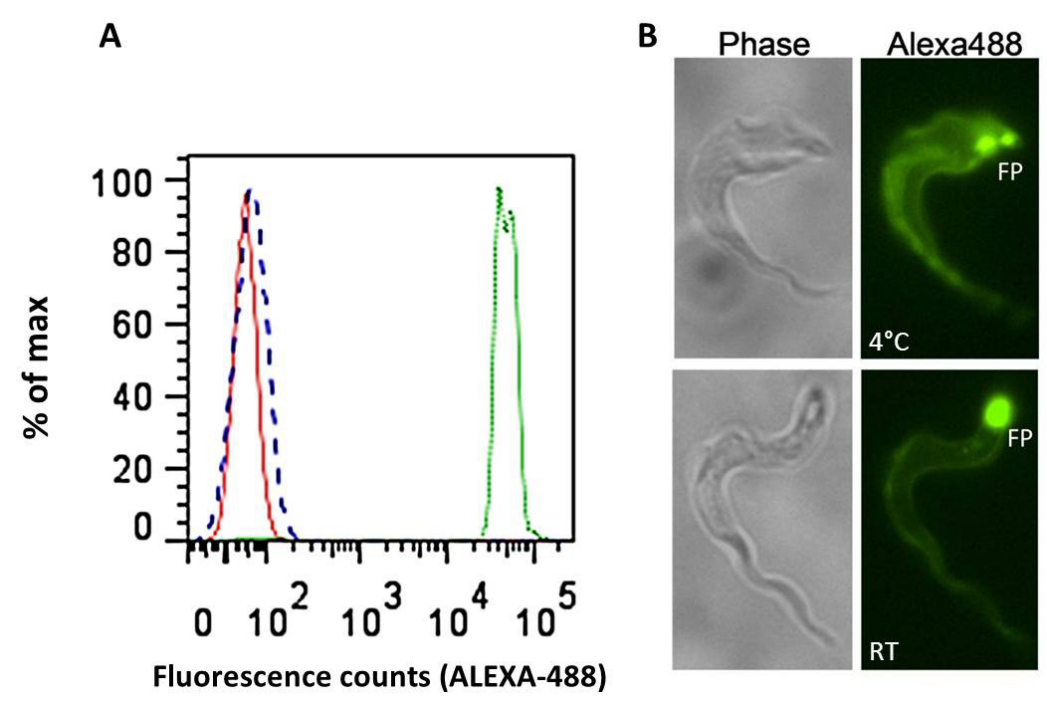
**Recognition of living trypanosomes expressing AnTat1.1 by ALEXA488-labelled Nb_An33 purified from *S. glossinidius***. A) Flow cytometry profile Red histogram: profile of parasites in the absence of Nbs. A non VSG specific Alexa488-labelled control nanobody did not significantly bind to the trypanosome surface (Blue dashed histogram). Green histogram: profile of parasites bound by Alexa 488-labelled Nb_An33 secreted by *Sodalis glossinidius*. B) Immuno-fluorescence microscopy. When maintained at room temperature surface bound Alexa488-labelled Nb_An33 gradually accumulated in the parasite flagellar pocket (FP).

The functionality of the extracellularly released Nb_An33 was further evaluated by analyzing its binding ability to living trypanosomes via flow cytometry and microscopy. *Sodalis *cells periplasmatically expressing Nb_An33 was grown in a 1.5 L liquid culture to an OD_600 _0.5. Nb_An33 was purified from the periplasm by immobilized metal affinity chromatography and gel filtration (Figure 5). The eluted fractions were pooled and concentrated on Vivaspin concentrators. Comparison of the size exclusion chromatograms of Nb_An33 produced by *Sodalis *versus *E. coli *reveals identical retention times. The purity of the concentrated eluded fractions was confirmed via immuno- and Coomassie staining (Figure 5, inset). Flow cytometry analysis showed that purified trypanosomes expressing AnTat1.1 VSG were stained upon the addition of Alexa488-labelled Nb_An33 (Figure 6A, green histogram). In the same experimental set-up, parasites were incubated with a control Alexa488-labelled Nb with no VSG specificity and the profile is indistinguishable from an unstained sample of parasites (Figure 6A, dashed and red histogram). Analysis of the samples by immunofluorescence microscopy showed a staining of the living parasites over their entire surface when incubated with Alexa488-labeled Nb_An33 at 4°C. However, during the microscopical analysis at room temperature (RT) the fluorescent labeled Nb accumulated mainly in the trypanosome flagellar pocket (FP) (Figure 6B). Together, these results provide definite evidence that Nb_An33 released by *S. glossinidius *is fully functional and able to target its specific epitope that is present in the dense VSG coat of living parasites.

## Discussion

Genetically modified bacterial symbionts of arthropod disease vectors are potential tools for the delivery of proteins that interfere with pathogen development in the vector and may serve as a powerful complementary approach to control disease transmission [2]. Furthermore, the use of bacterial symbionts expressing foreign proteins in disease-carrying arthropods has also an intriguing potential for studying insect-pathogen interactions. The advent of Nanobody^® ^technology has offered new prospects for the development of new effector molecules applicable for the paratransgenesis approach. These single-domain antigen-binding fragments represent exquisite targeting tools because of their small size (13-15 kDa) and stability properties [17,22]. Despite the interest for a paratransgenesis approach in tsetse flies to control transmission of African trypanosomiasis, little progress has been made on the identification and expression of trypanosome-interfering proteins in the tsetse fly bacterial endosymbiont *Sodalis glossinidius*. To date, S*. glossinidius *strains have only been used as hosts for the production of GFP [1]. In this study we explored the possibility of expressing a trypanosome-interfering Nanobody^® ^in *Sodalis glossinidius*. We have developed a suitable expression vector that allows for the expression in *S. glossinidius *of an anti-trypanosome Nanobody^®^, Nb_An33 that targets a high-mannose carbohydrate epitope present on the Variant-specific Surface Glycoprotein of *Trypanosoma brucei *[24].

Importantly, to control parasites in the tsetse fly, it is imperative for the effector molecules to reach their target. In an effort to address the need for an efficient secretion system we evaluated two distinct bacterial secretion pathways in their capacity to deliver Nbs to the extracellular environment. Nb_An33 fused to the *S. glossinidius *FliC secretion signal was expressed by both *S. glossinidius *and *E. coli*. However, in neither species could the fusion protein be detected in the culture medium nor the periplasm. The underlying reasons for this secretion failure remains unclear. One possibility includes the presence of a functional *Sodalis glossinidius *flagellar cap protein FliD, responsible for the polymerization of the FliC monomers into a filament, which would hinder the release of the FliC-fusion protein into the extracellular medium [26].

Nb_An33 was successfully exported to the *S. glossinidius *and *E. coli *periplasm using the pelB signal peptide. Furthermore, in both species the recombinant protein accumulated in the culture medium with an extracellular export efficiency in *Sodalis *of 20-25% during exponential and stationary growth phase where cell lysis is negligible. The extracellular release of small proteins and antibody fragments that were secreted into the periplasm via the pelB signal peptide has already been described [7,28]. The occurrence of this phenomenon appears to be highly dependent on the characteristics of the protein and is not yet fully understood. The secretion of some recombinant proteins to the periplasm is suggested to cause a destabilization of the outer membrane, which becomes leaky and results in the non-specific release of periplasmic proteins to the extracellular environment [29,30]. The pelB signal peptide is known to direct protein translocation to the periplasm via the Sec-dependent type II secretion pathway [31]. The feasibility of this pathway to export heterologous proteins to the periplasm of *S. glossinidius *complements the twin-arginine translocation (Tat) pathway, also involved with periplasmic transport, which was previously demonstrated to be functional in *Sodalis glossinidius *[32].

Additionally, the ELISA results indicated that during the stationary and cell lysis phase of the growth curve, *S. glossinidius *cells harboring the ppelBNb33*lac *plasmid released functional Nb_An33 in the culture medium where it accumulated at concentrations as high as 160 ng/ml. This clearly indicates that the release of Nbs from lysing *Sodalis *cells to the extracellular environment could also be an important way for the expressed Nb to reach its trypanosome target in the lumen of the tsetse fly. For both *S. glossinidius *strains harboring pNb33*lac* and pFliCNb33*fliC*, the amount active Nb_An33 expression was below the detection limit in ELISA, probably due to the fact that biological activity is dependent on correct protein folding involving the formation of disulfide bonds which is unlikely to occur in the reducing environment of the cytoplasm. The ELISA results also demonstrated that the secreted Nb_An33 was perfectly functional in terms of antigen binding to purified soluble AnTat 1.1 VSG *in vitro*.

Another important consideration when expressing potential effector proteins into the midguts of blood feeding arthropods is the susceptibility of the effectors to proteolytic degradation. Spiking of midgut extracts from tsetse flies with purified Nb_An33 in an ELISA assay demonstrated that Nb_An33 retains its antigen binding properties (unpublished results), providing preliminary evidence that Nb_An33 remains functional within the midgut environment of the fly. Furthermore, Nbs can easily be mutagenized and selected for increased proteolytic stability [33].

Finally, the capacity of the secreted Nb_An33 to recognize its epitope on living trypanosomes was confirmed by flow cytometry and fluorescence microscopy using alkaline binding conditions that are relevant for the tsetse fly midgut physiology.

Growth curve analysis and cell population doubling time of the *S. glossinidius *strains harboring the different expression constructs showed that there was a strong correlation between the ability of the *S. glossinidius *strains to export the Nb_An33 fusion protein and growth performance. Strains expressing Nb_An33 intracellularly showed a significant reduction in growth rate compared to the WT strains, while secreting strains were not affected in their growth. These results suggest that accumulation of Nb_An33 in the cytoplasm imparts a detrimental effect on growth performance and that efficiently secreting Nb_An33 to the periplasm rescues this effect, allowing the strain to grow with kinetics similar to the WT strain.

## Conclusions

This study provides the first demonstration of the functional expression and extracellular delivery of trypanosome-interfering proteins in *S. glossinidius*. Moreover, we demonstrated that *S. glossinidius *expressing pelBNb_An33 exhibited no significant reduction in terms of fitness, determined by *in vitro *growth kinetics, compared to the wild-type strain. This ability of the recombinant *S. glossinidius *strain to effectively compete with native strains is of great importance to the overall success of the paratransgenesis strategy. Given the ability of *S. glossinidius *to express high levels of active Nb_An33 and the capacity to release this anti-trypanosome Nb without hampering the bacterium viability, the foundation has been laid for further exploration of the inhibitory effect on trypanosome development in the tsetse fly. For this, highly potent trypanolytic Nbs have been developed very recently that lyse trypanosomes both *in vitro *and *in vivo *by interfering with the parasite endocytic pathway [34].

The current study also reinforces the notion for the potential of *S. glossinidius *to be developed into a paratransgenic platform organism. At a broad level, the concept of using Nbs as effector molecules to be delivered by bacterial endosymbionts is not limited to the tsetse fly-trypanosome model but could also be applied in a paratransgenic approach to encompass other vector-borne diseases.

## Methods

### Insects, bacterial strains and culture conditions

*Sodalis glossinidius *strains used in this study were isolated from the hemolymph of surface-sterilized *Glossina morsitans morsitans *from the colony maintained at the Institute of Tropical Medicine (Antwerp, Belgium). Cultures were maintained *in vitro *at 26°C in liquid Mitsuhashi-Maramorosch (MM) insect medium (PromoCell) supplemented with 10% (v/v) heat-inactivated fetal bovine serum (FBS). For cloning, *S. glossinidius *strains were cultivated on MM agar plates composed of MM medium (without FBS) solidified by autoclaving after the addition of 1% of bacto-agar. Blood agar plates were supplemented with 10% packed horse blood cells (IMP) and yeastolate (5 mg/ml) (Gibco). All solid cultures were cultivated in micro-aerophilic conditions generated using the Campygen pack system (Oxoid) which provided 5% O_2_, 10% CO_2_, balanced with N_2 _at 26°C. Where appropriate, antibiotics were added to the media at the following concentrations: 100 μg/ml of ampicillin and 50 μg/ml of kanamycin.

### Plasmids constructs

Plasmids used in this study and the structure of the fused genes are shown in Figure 1. *Nb_An33*, coupled to the *pelB *leader sequence and followed by a 6xHis tag was amplified as a *XbaI*-*EcoRI *fragment by PCR from the pHen6C plasmid containing the *pelBNb33 *gene using the following primer set: pelBNb33_FW, 5'-TTTTTCTAGAATGAAATACCTATTGCCTACGG-3' and Nb33_Rev, 5'- TTTTGAATTCTTAGTGATGGTGATGGTGGTGTGAGGAGACGGTGACCTG-3' (*XbaI*-*EcoRI *restriction sites are underlined). The resulting 438 bp PCR product was cloned into pCM66 [35] to create ppelBNb33*lac*. Nb33_FW, 5'-ATATTCTAGATGATGTGCAGCTGGTGGAGTC-3' was used to create pNb33*lac *without any secretion signal. To create pFliCNb33*fliC *and pFliCpelBNb33*fliC*, a 510 bp fragment including the *FliC *promoter region (*PfliC*) and a 210 bp fragment *FliC*, encoding 70 N-terminal amino acids of the *S. glossinidius FliC *gene, was inserted in-frame between the *SphI*-*XbaI *restriction sites of pNb33*lac and *ppelBNb33 respectively, with the following primer set: FliC_FW, 5'- GCATGCCATGTCCCAGGTCATT-3' and FliC_Rev, 5'-ATATCTAGAGTCATTGGCGCATG-3'(*SphI*-*XbaI *restriction sites are underlined). All plasmids were sequenced to confirm the desired DNA sequence and the correct reading frame.

### Genetic transformation of Sodalis glossinidius

Plasmid constructs were transformed in wildtype (WT) *S. glossinidius *cells using a heat-shock method [2]. Transformed cells were allowed to recover overnight at 26°C prior to plating onto MM-blood agar, supplemented with kanamycin and a single recombinant *S. glossinidius *colony was inoculated into liquid culture.

### Western Blot Analysis

Cultures were grown to the beginning of stationary phase (S*. glossinidius *OD_600 _0.5-0.6; *E. coli *OD_600 _1,5-2). Cells were pelleted from bacterial cultures by centrifugation (15 min, 10000 × g) and the supernatant was clarified from residual bacterial cells by a second centrifugation step. Proteins in the growth medium were precipitated with 10% trichloroacetic acid (TCA) for 1 h on ice. From the pelleted cells, periplasmic proteins were extracted by osmotic shock [36]. For SDS-PAGE, samples were heat denatured at 95°C in the presence of SDS-PAGE loading buffer containing β-mercaptoethanol and analyzed on a 12% (w/v) polyacrylamide gel. Proteins were transferred onto a nitrocellulose membrane (Whattman). After overnight blocking with 1% (w/v) bovine serum albumin, the membrane was incubated sequentially with a mouse anti-6xHis-tag IgG1 antibody (1:1000) (Serotec) and a rabbit anti-mouse-IgG antibody (1:1000) (Serotec) conjugated to horseradish peroxidase. In between these successive 2 h incubations, the membrane was washed with PBS-0.1% Tween 20. Thirty minutes after adding the substrate (TMB 1-Component Membrane Peroxidase Substrate, KPL) the reaction was stopped by washing the membrane with water.

### Growth curve measurements

Logarithmically growing cultures were used to inoculate 25 ml of MM-medium to an optical density at 600 nm (OD600) of 0.005. Cultures of the different *S. glossinidius *strains were allowed to grow without shaking for 48 h and samples were taken every 24 h. Then cultures were transferred to a shaking incubator and grown in alternating cycles (12 h) of static and shaking conditions. During the exponential growth rate 2 samples/day were taken. Doubling times during exponential growing phase were calculated using the following equation: doubling time (in hours) = h*ln(2)/ln(c2/c1) where c1 is the initial concentration and c2 is the concentration when cultures reached maximum densities.

### Analysis of fusion protein concentrations using ELISA

The amount of active Nb_An33 present in cytoplasmic extracts and growth medium was quantified using an optimized Nanobody Solid-phase Binding enzyme-linked immunosorbent assay (ELISA) [37]. Samples were taken at the same time points indicated for the growth curve measurements. At each time point, 1 ml of culture media was centrifuged two times (8000 × g) to obtain the extracellular and whole cell fractions. The whole cell extracts were prepared by resuspending the cell pellets in 0.2 ml PBS supplemented with protease inhibitor (Roche) followed by sonication at an amplitude of 10 μm for 5 s (3 cycles on ice).

Maxisorb 96-well plates (Nunc) were coated overnight (4°C) with 200 ng purified soluble AnTat 1.1 VSG (target of Nb_An33) per well in 0.1 M NaHCO_3_, pH 8.2. Residual protein binding sites were blocked for two hours at room temperature with 0.5% bovine serum albumin (BSA) in PBS. In order to quantify Nb_An33 present in cytoplasmic extracts and growth medium, a standard serial dilution series (1:2) starting from 2500 to 5 ng/ml of purified Nb_An33 was prepared in PBS and MM-medium respectively. MM medium and PBS alone were included as blanks. Standards, cytoplasmic and extracellular fractions were added for 1 h at room temperature. Detection of antigen-bound Nanobodies was performed with a mouse anti-6xHis IgG antibody (Serotec) directly conjugated to horseradish peroxidase. Thirty minutes after adding peroxidase substrate, the reaction was stopped with 0.33 M H_2_SO_4 _and the optical density was measured at 450 nm (690 nm was used as reference filter). Protein concentrations were calculated from a standard curve fitted to a four parameter logistic equation using the Ascent software (Labsystems).

### Parasites and VSG purification

Purification of *Trypanosoma b. brucei *AnTat1.1 soluble VSG was prepared as described earlier [23]. *Trypanosoma b. brucei *AnTat1.1 bloodstream parasites were grown in mice and purified from their heparinized blood by using diethylaminoethyl cellulose (DEAE) anion exchange chromatography on mAECT columns [38]. Mouse care and experimental procedures were performed under approval from the Animal Ethical Committee of the Vrije Universiteit Brussel, (VUB), Belgium (ethical clearance N° 09-220-08).

### Purification and ALEXA-488 labelling of Nb_An33 produced in Sodalis glossinidius

Large-scale production of *Sodalis *Nb_An33 was performed in 500 ml shake flasks by growing the bacteria in an orbital shaker at 200 rpm to an OD_600 nm _of approximately 0.5. After cells were pelleted, periplasmic proteins were extracted by osmotic shock [36] and medium was concentrated and extensively dialyzed (6-8 kDa MWCO Spectra/Por dialysis membrane, Spectrum laboratories) against PBS. Proteins were purified from the periplasmic fraction and medium using affinity chromatography on a Ni-NTA Superflow column (Qiagen). The eluted fractions were concentrated on Hydrosart Vivaspin concentrators with a molecular mass cutoff of 5 kDa (Vivascience). Further purification was performed by size-exclusion chromatography (AKTA explorer, GE Healthcare) using a Superdex75 (HR10/30) column equilibrated with PBS and the purity of the proteins was evaluated by Coomassie-stained 12% SDS-polyacrylamide gel and their identity confirmed by 6 × His tag specific immunodection in Western blot. ALEXA Fluor 488 labelling of the nanobody was accomplished using the Alexa Fluor^® ^488 Monoclonal Antibody Labeling Kit (Molecular Probes) according to the manufacturer's instructions. To separate the nanobody from free label, a second Superdex 75 (HR10/30) gel filtration chromatography was performed.

### Flow cytometry analysis and Immunofluorescent Microscopy

The binding capabilities of the labeled and purified Nb_An33 from *S. glossinidius *was evaluated on live, bloodstream form AnTat1.1 trypanosomes through flow cytometry. Aliquots of purified parasites (2 × 10^5 ^in 20 μl HMI-9 medium + 15% FCS, pH 8, [39]) were cooled on ice prior to adding ALEXA-labeled Nb_An33. After 30 min of incubation parasites were washed (8 min, 850 × g) with HMI9 medium to remove free label and analyzed in by flow cytometry on a FACS Canto II and histograms were prepared using the FlowJo software (Becton Dickinson, San Jose, CA). Immunofluorescence microscopy was performed on the same samples using a Nikon ECLIPSE E600 epifluorescence microscope equipped with a Plan Apo 60 × oil-immersion objective lens.

## Competing interests

The authors declare that they have no competing interests.

## Authors' contributions

Conceived and designed the experiments: LDV, GC, JVDA. Performed the experiments: LDV, GC. Contributed reagents/materials/analysis tools: BS, PDB, MC, JVDA. Wrote the paper: LDV, GC, JVDA, BS, PDB, MC. All authors read and approved the final manuscript.
